# Retraction: An immunoinformatics and extended molecular dynamics approach for designing a polyvalent vaccine against multiple strains of Human T-lymphotropic virus (HTLV)

**DOI:** 10.1371/journal.pone.0324071

**Published:** 2025-05-06

**Authors:** The PLOS One Editors

The *PLOS One* Editors retract this article [1,2] due to concerns about peer review integrity and potential manipulation of the publication process. These concerns call into question the validity of the reported results. We regret that the issues were not identified prior to the article’s publication.

MAU, NNI, MAK, and AMS agreed with the retraction. RBP did not agree with the retraction. TZ responded but expressed neither agreement nor disagreement with the editorial decision. ATM, NAR, TBR, NN, SIM, MNS, and NA either did not respond directly or could not be reached.
